# Immuno-virological response and associated factors amongst HIV-1 vertically infected adolescents in Yaoundé-Cameroon

**DOI:** 10.1371/journal.pone.0187566

**Published:** 2017-11-07

**Authors:** Joseph Fokam, Serge Clotaire Billong, Franck Jogue, Suzie Moyo Tetang Ndiang, Annie Carole Nga Motaze, Koki Ndombo Paul, Anne Esther Njom Nlend

**Affiliations:** 1 Virology Laboratory, Chantal BIYA International Reference Centre for research on HIV/AIDS prevention and management, Yaoundé, Cameroon; 2 Faculty of Medicine and Biomedical Sciences, University of Yaoundé I, Yaoundé, Cameroon; 3 National HIV Drug Resistance Prevention and Surveillance Working Group, Ministry of Public Health, Yaoundé, Cameroon; 4 Research, Planning, Monitoring and Evaluation Service, Central Technical Group, National AIDS Control Committee, Yaoundé, Cameroon; 5 National Social Insurance Fund Hospital, Paediatric Service, Yaoundé, Cameroon; 6 Mother-Child Centre, Chantal BIYA Foundation, Yaoundé, Cameroon; 7 Higher Institute of Medical Technology, Faculty of Medicine and Pharmaceutical Sciences, University of Douala, Douala, Cameroon; Universita degli Studi di Roma Tor Vergata, ITALY

## Abstract

**Introduction:**

Limited studies have reported the outcomes of lifelong antiretroviral therapy (ART) amongst adolescents living with HIV (ALWHIV) in resource-limited settings (RLS), thus classifying this population as underserved. We therefore aimed to ascertain the immunological and virological responses, and associated factors amongst Cameroonian ALWHIV.

**Method:**

A cross-sectional and observational study was conducted from January through May 2016 at the National Social Insurance Fund Health Centre in Yaoundé-Cameroon. Immunological and virological responses were evaluated using CD4 cell count and viral load respectively, with viral suppression (VS) defined as <50 copies/ml. Adherence was evaluated using self-reported missing doses during the past 14 days. Data were analyzed using R v.3.3.0, with p<0.05 considered statistically significant.

**Results:**

Of the 145 ALWHIV on ART enrolled in the study, 52% were female, median age [interquartile (IQR)] was 13 [11–16] years, median [IQR] time-on-ART was 7 [5–10] years, 48% were orphans, 92% were on first-line ART and 36% were adherent to ART. Following ART response, 79% (114/145) had CD4 ≥500/mm^3^, 71.0% (103/145) were on VS of whom 52.4% (76/145) had a sustained VS. Duration of ART was associated with immune restoration (Odd Ratio 3.73 [1.26–12.21]) but not with virological response. Risks of poor adherence were greater in orphans of both parents (p = 0.078).

**Conclusion:**

In this urban setting of Cameroon, ALWHIV receiving ART show favorable immunological and virological response in a medium run. For long-term ART success, implementing a close monitoring of adherence and risks of viral rebound would be highly relevant, especially for orphans of both parents.

## Introduction

Even though AIDS-related deaths has fallen by 35% globally from 2005 to 2013, deaths among adolescents living with HIV (ALWHIV) have sharply increased, reaching 50% [1]. Of note, in the era of antiretroviral therapy (ART), AIDS remains the second leading cause of death among adolescents worldwide and the leading cause of death among adolescents in sub-Saharan Africa (SSA) [1,2]. This high mortality is largely attributed to challenges in linking to and retaining in care ALWHIV, as well as appropriate transitioning from paediatric to adult ART regimens [1,2].

In order to ensure a better life expectancy as these children grow-up, the universal access to pediatric ART and related programmatic actions have been implemented, leading to an increasing number of ART-experienced children reaching adolescence even in resource-limited settings (RLS) [2–4].

However, in addition to healthcare linkage and retention on treatment, ALWHIV are facing other challenges in the continuum of care a long run, which include adherence to ART and compliance to caregivers [5]. Of note, levels of adherence can be easily reversed as the child grows up, especially in the frame of inappropriate disclosure of HIV status, orphan status, varying galenic formulations and other adolescence related issues [6–8].

An adequate management of ALWHIV would help in preserving the lifelong benefits of ART initiated since infancy and in defining an appropriate transition from pediatric to adult regimens, thereby limiting events of treatment failure and the advent of AIDS [8,9]. In addition to suboptimal adherence, complete treatment interruption is common during adolescent crisis and might lead to life threatened events such as opportunistic infections. Particularly, ART interruption appears to be common in ALWHIV with distinct familial features and risky lifestyles [5,6], mainly favored by the aforementioned inefficient disclosure of HIV status, inappropriate transition from pediatric to adult care, and the classical scenario of adolescent crisis when faced with chronic diseases [7–9].

In Cameroon, about 7,100 children and adolescents aged below 15 years were enrolled on ART by end of 2015, a number expected to rise with the ongoing universal access to ART for children nationwide [10]. Because the footprint of long-term ART success largely depends on individual and programmatic features [11,12], it becomes crucial to ascertain the breath of treatment response amongst ALWHIV, to delineate determinants of treatment outcomes, in order to design context-specific recommendations toward public health actions in settings like Cameroon. Such measures are of great relevance in the frame of scarcity of data reporting on health outcomes in the long-term management of ALWHIV [5–9].

Our study objectives were to evaluate immunological and virological responses on ART, adherence level, and their respective determinants amongst Cameroonian ALWHIV.

## Methods

### Study design

A cross-sectional study, nested in an observational cohort of ALWHIV, was conducted from January through May 2016 at the National Social Insurance Fund Health Centre of Essos, in the township of Yaounde, the capital city of Cameroon. This study site is a referral health facility for care and treatment of HIV infected children since 2005. Children followed up at the study site were all HIV-vertically infected, except for one that was infected through unsafe blood transfusion. None of the adolescent was behaviorally contaminated.

### Sampling method and eligibility criteria

Based on a consecutive sampling, the required minimum sample size for the study was calculated using the expected rate of virological failure (VF, between 6–20%) as provided by the WHO (World Health Organization) Pediatric ART optimization group [12], by using the following statistical formula: N = Z^2^xP(1-P)/D^2^.

With “Z” equal to 1.96 at 95% confidence interval (CI), with “P” considered as a median rate of VF (10%) [12], and “D” being the error rate set at 5% (0.05); the minimum sample size “N” = 138.3, rounded-up to a minimum of 139 participants to be enrolled in the study.

Eligibility criteria were every ALWHIV who: (a) is aware of his HIV status, (b) is registered for ART monitoring at the study site, (c) is receiving ART for at least six months, (d) is capable of responding to the study questionnaire, and (e) has provided a written consent.

### Clinical and laboratory procedures

During routine clinic attendance at the study site, a standard questionnaire was administered to all ALWHIV, covering socio-demographic data, family status, treatment history and adherence level, as well as basic clinical and biological parameters. Using the self-reported approach for adherence assessment, poor adherence was defined as the number of missing doses during the last 14 days. The study questionnaire (data collection sheet), in both French and English language, has been included as supplemental digital content *S1 File*.

CD4 cell count was performed using the BD FACS Count system as per the manufacturer’s instructions (https://www.bdbiosciences.com/documents/BD_FACSCount_Brochure.pdf). Viral load measurement was performed using the Abbott Applied Biosystem m2000RT Real Time PCR AB m2000RT as-per the manufacturer’s instructions (*Abbott Laboratories*, *USA*), with a lower detection threshold of 40 HIV-1 RNA copies/ml and an upper detection threshold of 10,000,000 copies/ml (www.abbottmolecular.com/products/infectious-diseases/realtime-pcr/hiv-1-assay).

### Data interpretation

Primary outcomes from the study were the rate of ALWHIV having a normal immune status and the rate of ALWHIV experiencing viral suppression (VS). A normal immune status was defined as absolute CD4 ≥500 cells/mm^3^, while VS was defined a viral load <50 HIV-1 RNA copies/ml. Sustained VS was defined as two consecutive viral load <50 HIV-1 RNA copies/ml. Adherence was evaluated by self-reporting, and poor adherence was defined as missing ≥ one dose of ART during the past 14 days.

### Statistical analysis

Dependent variables were normal immune status and VS, while independent variables were duration of ART, type of ART regimen, gender, family status, and adherence level. Fisher exact test and Mann-Whitney tests were used to analyze qualitative and quantitative variables respectively. Univariate and multivariate regression were used for covariates with immunological status and VS; only p-values >0.2 in univariate analyses were considered in regression analysis. Statistical analyses were performed using R v.3.3.0, with p<0.05 considered statistically significant.

### Ethical considerations

Administrative authorisation was issued and ethical clearance for the study was obtained from the Institutional Review Board (IRB) of the *Essos* Health Centre (Reference: *Nº2016/09/CE-CHE*). Mothers/guardians and eligible adolescents received detailed information on the study (with provision of an information sheet). Compliant mothers/guardians and adolescents aged of ≥ 13 years then provided each a signed written proxy-informed consent; in addition to assent received from each ALWHIV aged <13 years. The consent procedure was approved by the IRB. Laboratory results of viremia and CD4 cells count were freely delivered to each participant for their clinical benefits, data management was under strict confidentiality by using unique identifiers and access to data is protected by an encrypted password.

Study dataset is provided as supplemental digital content *S2 File*

## Results

### Socio-demographic and clinical characteristics of the population

A total of 145 ALWHIV were enrolled in the study. Amongst them, 52% were females, the median age [interquartile range, IQR] was 13 [11–16] years, and almost all appeared clinically asymptomatic. About 50% of them were orphans of single or both parents.

The median duration [IQR] on ART was 7 [5–10] years, 92% were on first-line ART regimen consisting of two nucleoside reverse transcriptase inhibitors (NRTIs) and one non-NRTI (NNRTI), and 36% were considered adherent (Table 1).

**Table 1:** Characteristics of the study population.

Study population of ALWHIV	N	%
**Gender**		
Male	70	48.28
Female	75	51.72
**Sustained viral suppression**		
Yes	76	52.41
No	69	47.59
**Immunological status**		
<500 CD4/mm^3^	31	21.38
>500 CD4/mm^3^	114	78.62
**Missing doses (n = 144)**		
** Yes**	92	63.89
** No**	52	36,11
**Participant family profile**		
Orphan by father	17	11.72
Orphan by mother	27	18.62
Orphan of both parents	26	17.93
Non-orphan	75	51.72

**Legend**. ALWHIV: adolescents living with HIV; CD: clusters of differentiation.

### Immunological status and associated factors

At the threshold of 500 CD4/mm^3^, 79% of ALWHIV had a normal immune status, indicating a possible immune recovery with ART. In bivariate analysis, immune profile was neither associated to gender, nor to ART history (the type of ART regimen, the duration of ART, ART switch, or missing doses of ART). However, normal immune status appeared to be higher amongst non-orphan ALWHIV (Table 2).

**Table 2:** Determinants of immune response.

	OR Non-adjusted [IC 95%]	OR adjusted [IC 95%]
Gender of ALWHIV		
Male	Ref	Ref
Female	1.64 [0.69, 4.03]	2.03 [0.84, 5.14]
**Participant family profile**		
Orphan by father	Ref	Ref
Orphan by mother	1.54 [0.33, 7.00]	1.8 [0.42, 7.74]
Orphan of both parents	1.22 [0.27, 5.36]	1.21 [0.28, 5.15]
Non-orphan	3.48 [0.86, 13.42]	3.63 [0.93, 13.81]
**Treatment change**		
Yes	Ref	Ref
No	0.91 [0.20, 3.14]	1.08 [0.26, 3.72]
**ART regimen**		
1^st^ line	Ref	Ref
2^nd^ line	0.34 [0.086,1.48]	0.26 [0.058, 1.17]
**Missing doses**		
Yes	Ref	Ref
No	1.06 [0.43, 2.70]	0.91 [0.265, 3.71]
**Duration on ART (years)**		
(1,5]	Ref	
(5,9]	2.59 [0.92, 7.81]	3.73 [1.26, 12.21]
(9,16]	1.95 [0.65, 6.38]	3.98 [1.09, 16.6]

**Legend**. ALWHIV: adolescents living with HIV; ART: antiretroviral therapy.

In multivariate analysis, a better immune status appeared to correlate with the duration of ART treatment (Odd ratio 3.7 [1,3–12,2]) amongst ALWHIV having more than 5 years of ART, as compared to those treated for less than 5 years, an observation that was sustained even for those ALWHIV on treatment for more than 9 years (Table 2).

### Viral profile and associated factors

Using a threshold of ≥ 1,000 HIV-1 RNA copies/ml as considered by the WHO for defining VF, 20.7% (30/145) of ALWHIV were experiencing VF. Using the optimal threshold for VS of <50 HIV-1 RNA copies/ml, 71.0% (103/145) had an undetectable viral load at the last measurement, with a history of sustained VS (i.e. <50 copies/ml) reported in 52.4% (76/145). A proportion of 8.3% (12/145) ALWHIV had a detectable low-level viremia (between 50–999 copies/ml), which was neither classified as VS nor VF.

Rates of VF and of missing doses of ART appeared more important among orphans than those having their parents. However, virological response was neither associated with the duration of ART, nor with the type of ART regimen, adherence level and even the gender (Tables 3 and 4).

**Table 3:** Determinants of virological response.

	Virological failure N = 30	Undetectable viral load N = 103	Detectable viral load N = 12	P-value
Gender of ALWHIV				**0.40**
Male	17	49	4	
Female	13	53	8	
**Participant family profile**				**0.04**
Orphan by father	3	11	3	
Orphan by mother	9	17	1	
Orphan of both parents	8	13	5	
Non-orphan	10	61	3	
**Treatment change**				**0.92**
Yes	4	14	2	
No	26	88	10	
**ART regimen**				**0.078**
1^st^ line	26	98	8	
2^nd^ line	4	4	4	
**Duration on ART (years)**				**0.59**
(1,5]	14	33	4	
(5,9]	9	40	6	
(9,16]	7	29	2	
**Poor adherence (missing doses)**				**0.95**
Yes	20	64	8	
No	10	38	4	

**Legend**. ALWHIV: adolescents living with HIV; ART: antiretroviral therapy.

**Table 4:** Predictors of poor adherence (missing ARV doses).

	OR adjusted	p-value
**Age**		
10–12	Ref	Ref
12–14	1.0869923	0.8696
14–19	0.5959906	0.2684
**Viral load classification**		
Virological failure	Ref	Ref
50–1000 copies/ml	0.7647322	0.7320
Undetectable	1.1332441	0.7945
**Gender**		
Male	Ref	Ref
Female	1.2375570	0.5627
**Participant family profile**		
Orphan by father	Ref	Ref
Orphan by mother	0.7049898	0.5913
Orphan of both parents	1.2613368	0.6435
Non-orphan	2.5431368	0.0777

**Legend**. ARV: antiretroviral.

## Discussion

The present study addresses the outcomes of ALWHIV receiving ART in an urban setting in Cameroon, with the goal to provide evidence for sustained VS in this target population living in RLS.

After a median of seven years of ART, ALWHIV could still be on a successful first-line regimen in RLS like Cameroon. With the median age of 13 years in a population apparently asymptomatic, these findings also demonstrate the benefits of ART amongst ALWHIV, and advocate for strategies toward improved management [12–14].

Regarding the immune status, about 8 out of 10 ALWHIV might have a normal immunity, with a significant immune reconstitution expected over years of ART experience (about 4 folds beyond five years). Our findings therefore support the concept of slow immune recovery on ART, in spite of VS during the first year of ART initiation [15,16]. This observation confirms CD4 as a biomarker useful to evaluate immune response only after long-term ART exposure. Interestingly, a better immune recovery was observed amongst girls as compared to boys, likely due to higher compliance to ART. In addition, immune response was very poor amongst orphans of both parents, indicating the importance of parent/guardian in the continuum of adolescent care and the need for close monitoring of this target population, by implementing special adherence educational programs [17–18]. As ART regimen (defines as first or second-line ARV drug combination) does not have a considerable impact on immune response locally, focusing on type of regimen to predict treatment response/outcomes might not be necessary in similar settings [19].

With 71% of VS (i.e. viral load <50 copies/ml) and about 20% of VF, after a median of seven years on ART in this urban setting, it clearly appeared that ALWHIV might be responding favorably to current treatment. However, in a frame where sustained VS remains challenging, it would be relevant to monitor those with a sustained low-level viremia (~8% in our findings) in order to overcome on-going viral replication and limit the risk of drug resistance emergence [9,11]. As the majority of ALWHIV are still receiving a first-line ART regimen, it appears current guidelines are still effective, and their effectiveness should be further sustained by close adherence monitoring, parental/guardian support, and timely viral monitoring.

With about 80% of sustained viremia below 1,000 copies/ml, additional efforts are required to meet the third pillar of the UNAIDS 90-90-90 target amongst ALWHIV [12,13], by emphasizing on factors promoting constant adherence as aforementioned (boys in general, and orphans of both parents in particular) [13,20–22]. On one hand, similar reports were shown in Asia [17,18], with favorable response despite delayed ART initiation and the use of drugs with low genetic barriers in some SSA settings [19–21]. On the other hand, our findings are far beyond previous reports from other SSA settings (50% VF, and poor immune recovery likely attributed to shorter observational periods) [15,16]. Globally, varying levels of VS 27-(89% VS after 2–5 years on treatment) and immune recovery have been reported amongst ALWHIV, thus supporting a context dependent outcome [22–24]. Thus, defining specific interventional programs, according to local realities/challenges, would greatly improve ART response amongst ALWHIV, and ensure adequate transition from into adult regimens/services in such RLS [24–26].

Our study limitations could be the cross-sectional design (inability to assess the impact of interventions, including health educational programs), the lack of a better adherence assessment tool, and the lack of HIV drug resistance testing amongst ALWHIV experiencing VF. Further studies are therefore needed to delineate non-adherent ALWHIV from those with a real VF, those requiring a treatment switch due to viral resistance, as well as other behavioral factors potentially associated with poor treatment outcomes. Nonetheless, the rate of detectable viremia serves as a warning indicator to stakeholders (ART managers and staffs involve in adherence counselling and therapeutic education) and paves the way for sustained viral control as a key to a successful transition or transfer from pediatric to adult care.

## Conclusion

In this urban RLS, adolescents perinatally infected with HIV exhibit a favorable immune and viral response after a medium-term on treatment. Specific interventions, including psychosocial supports, targeting mainly boys and orphans, would greatly improve adherence, sustained VS and immune restoration, for a successful transition to adult care.

## Supporting information

S1 File Questionnaire immuno-virological response adolescents. (PDF)

S2 File CD4-PVL-response-adolescents-Cameroon. (PDF)
