# Supplementary material for: Immuno-virological response and associated factors amongst HIV-1 vertically infected adolescents in Yaoundé-Cameroon
Source: PLoS One. 2017 Nov 7;12(11):e0187566. doi: 10.1371/journal.pone.0187566 (PMC5675438; doi:10.1371/journal.pone.0187566)
Supplement: S1 File — (PDF) [file pone.0187566.s001.pdf]

S1 FILE

QUESTIONNAIRE FOR ASSESSMENT OF IMMUNO-VIROLOGICAL RESPONSE ADOLESCENTS

DATA COLLECTION SHEET

Identification

Name: Telephone N°:

Surname: Identification code N°:

I) Socio-demographic features

Sex: Male ☐ Female ☐

Age: _____years

Parents' survival status:

- Orphan of father: 1
- Orphan of mother: 2
- Orphan of both parents: 3
- Non-orphan: 4

II) Anthropometric data

Weight:______kg **Height** : _____metre

III) Biomedical data

Age at ARV treatment initiation:_____ years ; ARV regimen: ____________________

Change of treatment regimen during monitoring:__________________ Date________________

Number of viral load tests performed:_________ Number of CD4 tests performed:_________

Current viral load:_________ Current CD4 count:________

Sustained viral suppression: YES (1); NO (2)

WHO clinical stage: I ___; II____; III____; IV____

	Date	CD4 count (cell/mm^3)	CD4 count (%)	Viral load (absolute value)	Viral load (logarithm)
**History of immunological and virological parameters**	__ / __ / ____				
	__ / __ / ____				
	__ / __ / ____				
	__ / __ / ____				
	__ / __ / ____				
	__ / __ / ____				
	__ / __ / ____				
	__ / __ / ____				

Adherence to antiretroviral treatment during the last 14 days:

Statements	Yes	No
Have you ever missed your dosage during these last 14 days?		

FICHE DE COLLECTE DES DONNEES

Identification

Nom :　　　　　　　　　　　　　　N° de téléphone :
Prénom :　　　　　　　　　　　　　N° d'anonymat :

I) Données sociodémographiques

Sexe : Masculin ☐　　　　Féminin ☐

Âge : _____ans

État de survie des parents :
- Orphelin de père : 1
- Orphelin de mère : 2
- Orphelin des deux parents : 3
- Non orphelin : 4

II) Données anthropométriques

Poids :______kg　　　　**Taille** : _____mètre

III) Données biomédicales

Age à l'initiation du traitement ARV:_____ ans ; Protocole ARV : ___________________
Changement de protocole au cours du suivi :__________________ Date_______________
Nombre de charge virale : _________　　　Nombre de CD4 :__________
Charge Virale actuelle :_________　　　Taux de CD4 actuel :________
Suppression virale permanente : OUI (1);　　　NON (2)
Stade clinique OMS: I ___; II____; III____; IV____

	Date	CD4 (cell/mm^3)	CD4 (%)	Charge virale (valeur absolue)	Charge virale (logarithme)
**Historique des paramètres immunologiques et virologiques**	__ / __ / ____				
	__ / __ / ____				
	__ / __ / ____				
	__ / __ / ____				
	__ / __ / ____				
	__ / __ / ____				
	__ / __ / ____				
	__ / __ / ____				

Observance du traitement antirétroviral durant les 14 derniers jours:

Libellés	Oui	Non
Avez-vous eu a sauté la prise de médicaments durant ces 14 derniers jours ?		
