# Supplementary material for: Immuno-virological response and associated factors amongst HIV-1 vertically infected adolescents in Yaoundé-Cameroon
Source: PLoS One. 2017 Nov 7;12(11):e0187566. doi: 10.1371/journal.pone.0187566 (PMC5675438; doi:10.1371/journal.pone.0187566)
Supplement: S2 File — (PDF) [file pone.0187566.s002.pdf]

S2 table -title : Data set of CD4 cell and viral measures in adolescents on antiretroviral therapy , Cameroon).

DICO_ID	AGE	SEX	Parental Survival	Weight	Height	Year of ART Start	AGE AT START in years	Story of Change in protocol	Year of protocol change	Number of Viral load measures	Current viral load	ABSOLUTE CD4 CELL COUNT	PERMANENT VIRAL SIPPRESSION	viral resupression	Missing doses of ART
1	12	male	2	36	146	2011	7	2	NA	2	0	693	1	2	1
2	16	male	1	62	176	2005	4	2	NA	10	0	900	2	1	1
3	12	Female	2	38	149	2005	1	2	NA	3	0	1404	1	2	1
4	12	male	4	38	142	2007	3	2	NA	10	0	1141	1	2	2
5	11	male	2	28	132	2011	6	2	NA	2	0	1043	2	1	1
6	10	Female	2	42	158	2009	3	2	NA	7	5302	844	2	1	1
7	11	Female	4	25	132	2013	8	2	NA	0	NA	576	1	2	2
8	12	Female	1	31	137	2004	0	2	NA	6	0	806	1	2	2
9	11	male	4	33	142	2005	0	1	2008	11	0	1180	2	1	1
10	12	male	2	32	134	2014	10	2	NA	6	0	1155	1	2	2
11	11	male	1	39	136	2008	3	2	NA	4	0	1254	2	1	1
12	11	male	4	26	133	2006	2	2	NA	7	213	1254	2	1	1
13	17	Female	3	44	147	2009	10	2	NA	6	0	998	2	1	1
14	13	Female	4	43	151	2008	5	2	NA	7	0	1216	1	2	1
15	10	Female	2	35	127	2007	0	2	NA	5	0	2134	1	2	1
16	17	male	2	49	146	2009	10	1	2013	5	427	958	2	1	1
17	19	male	3	42	150	2006	9	2	NA	9	0	817	2	1	1
18	13	Female	4	56	145	2011	8	2	NA	4	0	632	1	2	2
19	11	Female	4	28	134	2011	6	2	NA	4	0	1932	1	2	2
20	12	Female	1	39	140	2009	5	2	NA	3	0	1862	1	2	1
21	10	Female	2	25	125	2014	8	2	NA	1	30123	134	2	1	1
22	14	Female	2	41	142	2006	4	1	2009	9	59512	412	2	1	2
23	13	Female	2	27	132	2012	9	2	NA	2	0	1192	1	2	2
24	15	Female	4	55	156	2009	8	2	NA	6	0	680	2	1	1
25	14	Female	2	45	154	2004	0	1	2009	10	0	628	2	1	1

26	11	male	2	30	135	2011	6	2	NA	3	90360	1012	2	1	1
27	16	Female	4	61	160	2009	9	2	NA	7	0	948	1	2	2
28	16	male	2	41	151	2013	13	2	NA	4	0	1178	1	2	1
29	13	Female	4	42	146	2013	10	2	NA	1	0	587	1	2	2
30	13	Female	4	41	147	2011	8	2	NA	4	0	898	1	2	1
31	14	Female	4	36	140	2004	2	2	NA	7	0	628	1	2	2
32	15	male	4	44	158	2011	10	2	NA	9	0	978	1	2	2
33	17	Female	4	50	143	2003	5	1	2009	12	0	987	2	1	1
34	18	male	4	62	164	2006	8	2	NA	12	0	634	2	1	2
35	12	male	4	40	150	2003	0	2	NA	7	0	1287	1	2	1
36	15	male	1	52	168	2005	4	2	NA	11	0	490	1	2	1
37	15	male	4	56	169	2004	3	2	NA	6	0	1148	1	2	1
38	11	male	4	38	135	2005	0	2	NA	7	0	859	1	2	2
39	15	male	4	47	151	2006	5	1	2007	6	8942	461	2	1	1
40	13	Female	2	45	151	2010	7	2	NA	3	3000	524	2	1	1
41	13	male	4	46	150	2008	8	2	NA	10	0	669	2	1	1
42	16	male	1	55	172	2004	4	1	2009	12	138556	422	2	1	1
43	11	Female	4	57	144	2005	0	2	NA	8	0	896	1	2	2
44	17	Female	4	35	128	2014	15	2	NA	3	93274	500	2	1	2
45	14	male	4	32	139	2011	9	2	NA	1	5648	553	2	1	2
46	14	male	4	33	136	2007	5	1	2009	4	0	1086	2	1	1
47	12	Female	1	40	147	2014	9	2	NA	2	0	470	1	2	2
48	14	Female	2	34	141	2007	5	2	NA	3	0	462	2	1	1
49	17	Female	3	59	152	2014	15	2	NA	1	302	353	2	1	1
50	15	male	2	48	160	2011	11	2	NA	5	0	457	2	1	1
51	13	male	4	44	154	2009	6	2	NA	4	0	1176	2	1	2
52	16	Female	4	54	148	2010	10	2	NA	3	1062	867	2	1	1
53	12	Female	4	45	149	2009	5	2	NA	3	0	1248	1	2	1
54	11	Female	2	23	138	2012	7	2	NA	3	0	896	1	2	2
55	13	male	4	41	138	2011	8	2	NA	4	0	714	1	2	1
56	10	Female	3	28	122	2015	9	2	NA	2	0	488	1	2	2
57	15	male	1	51	162	2004	3	2	NA	7	0	941	1	2	1
58	11	Female	4	36	131	2014	8	2	NA	2	1140	462	2	1	1

59	12	Female	4	33	138	2008	4	1	2010	10	78	669	2	1	1
60	19	Female	3	54	142	2015	18	2	NA	2	134	669	1	2	2
61	15	Female	4	48	154	2008	7	2	NA	10	0	1192	1	2	1
62	13	Female	4	30	152	2012	9	2	NA	2	0	1302	1	2	2
63	16	male	3	50	148	2007	7	1	2008	8	0	709	2	1	1
64	10	Female	4	31	124	2013	7	2	NA	2	0	645	1	2	1
65	11	Female	4	27	130	2013	8	2	NA	2	0	967	1	2	1
66	13	Female	4	34	140	2013	10	2	NA	2	0	552	1	2	1
67	12	Female	4	26	132	2009	5	1	2014	2	0	515	2	1	1
68	13	Female	4	39	142	2014	11	2	NA	2	0	905	1	2	2
69	13	male	1	39	122	2010	7	2	NA	4	0	1525	1	2	1
70	12	male	2	26	135	2015	11	2	NA	1	651418	46	1	2	2
71	14	Female	4	47	140	2002	0	1	2008	12	0	1342	2	1	1
72	16	male	3	46	154	2015	15	2	NA	1	5148	306	1	2	1
73	18	male	4	66	168	2009	11	1	2011	12	0	908	2	1	1
74	18	Female	4	50	153	2012	14	2	NA	4	0	1175	1	2	2
75	13	Female	4	33	145	2010	7	2	NA	8	0	984	1	2	1
76	11	Female	3	36	140	2011	6	2	NA	3	248	883	2	1	1
77	14	male	1	36	145	2011	9	2	NA	2	0	763	1	2	1
78	12	male	4	30	137	2008	4	2	NA	6	0	911	1	2	1
79	14	male	4	48	142	2009	7	2	NA	4	0	480	1	2	1
80	17	male	3	56	158	2010	11	2	NA	5	27699	579	2	1	1
81	14	male	3	32	135	2004	2	1	2009	11	0	656	2	1	2
82	13	Female	1	29	130	2009	5	2	NA	5	546	965	2	1	2
83	17	Female	3	55	157	2014	15	2	NA	3	26590	193	2	1	2
84	10	male	4	36	128	2007	1	2	NA	8	0	1117	1	2	1
85	17	male	3	43	161	2009	10	2	NA	6	0	1012	1	2	2
86	15	male	1	33	137	2007	6	2	NA	8	352	368	2	1	1
87	17	Female	3	68	165	2000	1	2	NA	9	143	786	2	1	1
88	17	Female	3	40	152	2012	13	2	NA	2	0	821	1	2	2
89	11	Female	3	30	136	2011	6	2	NA	3	0	976	1	2	2
90	17	Female	3	63	160	2001	3	2	NA	8	4855	621	2	1	1
91	11	male	3	32	141	2006	1	2	NA	6	0	864	1	2	2

92	11	male	4	32	151	2009	4	2	NA	4	0	1140	1	2	2
93	10	Female	4	21	117	2012	6	2	NA	7	0	1900	1	2	1
94	10	Female	3	26	117	2008	2	2	NA	5	0	1086	1	2	1
95	13	Female	4	34	143	2009	6	1	2014	4	0	857	2	1	1
96	14	Female	4	47	156	2011	9	2	NA	5	0	746	1	2	1
97	11	male	2	25	123	2011	6	2	NA	2	90360	1012	2	1	2
98	12	Female	4	32	147	2011	7	1	2013	3	0	549	2	1	2
99	19	male	3	64	178	2003	6	1	2015	12	350962	456	2	1	2
100	11	Female	2	38	132	2009	4	2	NA	5	0	1649	1	2	2
101	16	male	4	44	156	2003	3	1	2004	12	0	1692	2	1	1
102	16	male	2	46	162	2007	7	2	NA	9	13614	12	2	1	1
103	16	Female	4	51	144	2005	5	2	NA	6	0	776	2	1	2
104	15	Female	1	40	150	2007	6	2	NA	4	5489	322	2	1	1
105	19	male	3	61	172	2004	7	2	NA	12	33956	441	2	1	2
106	13	male	1	33	140	2009	6	2	NA	3	1218	435	2	1	1
107	18	male	4	50	164	2009	11	2	NA	3	0	904	2	1	1
108	18	Female	2	56	163	2008	10	2	NA	7	0	1103	2	1	1
109	11	male	4	44	156	2005	0	2	NA	12	708	879	2	1	2
110	15	Female	3	36	152	2014	13	2	NA	2	28778	309	1	2	1
111	16	male	4	58	171	2006	6	2	NA	5	0	1117	1	2	1
112	11	male	4	25	115	2015	10	2	NA	2	0	432	1	2	2
113	10	Female	2	26	123	2013	7	2	NA	2	0	989	1	2	2
114	10	male	4	23	129	2007	1	2	NA	6	3992	443	2	1	2
115	16	male	4	43	148	2013	13	2	NA	3	202000	10	2	1	1
116	12	Female	4	42	146	2004	0	2	NA	8	0	1084	1	2	1
117	15	Female	1	45	156	2007	6	2	NA	10	0	563	1	2	2
118	15	Female	1	39	147	2008	7	2	NA	6	131	546	2	1	1
119	15	male	2	48	159	2009	8	2	NA	5	0	1069	1	2	2
120	11	Female	4	31	148	2014	9	2	NA	2	2049	368	2	1	1
121	12	male	2	31	141	2009	5	2	NA	5	0	886	1	2	2
122	10	male	4	27	130	2009	3	2	NA	8	0	991	1	2	1
123	13	male	4	34	141	2010	7	2	NA	3	0	516	2	1	1
124	10	Female	2	31	132	2009	4	2	NA	6	0	608	1	2	1

125	17	Female	4	76	171	2015	16	2	NA	2	0	577	1	2	1
126	16	male	4	50	162	2008	8	2	NA	3	0	865	1	2	1
127	16	male	4	37	148	2004	4	2	NA	7	30244	183	2	1	1
128	16	male	4	41	156	2009	9	2	NA	8	480333	406	2	1	2
129	16	male	4	50	156	2010	10	2	NA	6	0	715	1	2	1
130	16	Female	4	45	149	2006	6	2	NA	6	0	1179	2	1	1
131	18	Female	3	48	151	2006	8	2	NA	5	0	1171	2	1	2
132	14	male	2	33	143	2013	11	2	NA	4	12375	475	2	1	1
133	16	Female	4	55	171	2006	3	1	2007	6	0	595	2	1	1
134	18	male	1	54	172	2014	16	2	NA	2	0	976	1	2	1
135	10	Female	4	20	122	2007	1	2	NA	4	0	1524	1	2	1
136	11	Female	4	33	143	2007	2	2	NA	3	0	1024	1	2	1
137	17	Female	4	52	168	2006	7	2	NA	6	0	1175	1	2	2
138	15	Female	3	54	149	2011	11	2	NA	3	176360	41	2	1	1
139	17	male	4	46	166	2004	5	2	NA	9	0	868	1	2	1
140	11	male	4	28	130	2007	2	2	NA	4	0	984	1	2	2
141	12	male	4	44	152	2004	0	1	2007	11	0	785	2	1	1
142	10	male	4	24	120	2007	1	2	NA	7	0	1154	1	2	1
143	18	Female	3	52	150	2014	17	2	NA	2	177	565	1	2	2
144	15	male	3	32	152	2007	6	2	NA	4	0	817	2	1	1
145	11	male	3	29	126	2012	7	2	NA	2	0	1442	1	2	2
